# Prevalence and related factors of caregiving burden among family caregivers of patients with mental illness in China

**DOI:** 10.3389/fpsyt.2025.1744877

**Published:** 2026-01-16

**Authors:** Ruibo Deng, Yongming Wu, Shuyun Huang, Yuhang He, Chenyu Liu, Ziyun Zhang, Kai Wu, Lam Mei Fong, Fengchun Wu, Hehua Li

**Affiliations:** 1 Clinical Medicine, Guangzhou University of Chinese Medicine, Guangzhou, China; 2 Clinical Medicine, Nanjing Medical University, Nanjing, China; 3 The Affiliated Brain Hospital, Guangzhou Medical University, Guangzhou, China; 4 School of Biomedical Sciences and Engineering, South China University of Technology, Guangzhou, China; 5 Psychiatric Service of the Centro Hospitalar Conde de São Januário, Macao, Macao SAR, China; 6 Guangdong Engineering Technology Research Center for Translational Medicine of Mental Disorders, Guangzhou, China

**Keywords:** burden, family caregivers, mental illness, prevalence, related factors

## Abstract

**Background:**

Family caregivers are essential yet often overburdened in mental healthcare systems. However, the burden of Family caregivers in Mainland China remains limited. This study examines the prevalence of caregiving burden among family caregivers of individuals with mental illness in Mainland China and explores its association with personal and familial factors.

**Methods:**

Employing a cross-sectional design, we recruited 400 family caregivers from the Affiliated Brain Hospital of Guangzhou Medical University and the Second Affiliated Hospital of Guangzhou Medical University in China. This study collected data using the Zarit Burden Interview (ZBI) and the Family Burden Scale of Disease (FBSD).

**Results:**

The prevalence of caregiving burden was 57.25%. Caregivers with burden reported higher levels of economic burden, greater disruptions in family activities and entertainment, increased family relationship tension, and worse physical and mental health among family members than those without burden. They also had longer caregiving durations, greater economic burden pressure due to the patient’s illness, and generally lacked co-caregivers compared to caregivers without burden. The impact of the patient’s illness on economic burden, family activities, family entertainment, and the physical and mental health of family members was positively associated with the occurrence of caregiving burden, while the presence of co-caregivers had a protective effect (OR < 1). ROC curve analysis further identified disruptions in family activities, family entertainment, and economic burden as significant predictors of caregiving burden (AUC > 0.70).

**Conclusions:**

Caregiving burden is strongly linked to economic burden, impaired family functioning, and the physical and mental health of family members, while co-caregiver support mitigates this burden. These findings highlight the need for comprehensive support strategies, including economic assistance and shared-caregiving models, to alleviate caregiver burden within the mental healthcare system.

## 1 Introduction

According to the World Health Organization, mental illness represents a major global public health challenge, with approximately 970 million people affected worldwide, accounting for 13% of the total population (1). Beyond its well-documented effects on patients’ physical and mental health, mental illness imposes considerable caregiving burden on caregivers, significantly increasing their vulnerability to anxiety disorders and depression (2). The ongoing paradigm shift in mental healthcare delivery has further amplified the crucial role of families in patient support systems (3). While caregivers provide essential assistance with daily living activities and enhance treatment compliance, the chronic stress associated with caregiving frequently precipitates mental health complications in caregivers themselves (4). Empirical evidence reveals high stress levels in this population, with 72% reporting severe stress, including 25% meeting the clinical criteria for depression and 29% exhibiting anxiety disorders (5). Therefore, addressing the caregiving burden of mental illness caregivers is of great significance.

Comparative studies show that caregivers of individuals with mental illness face significantly higher mental health risks than the general population (6, 7). While the prevalence of depression stands at 4.3% in community samples (8), rates among mental health caregivers vary dramatically, ranging from 19% to 57.7% (9). Notably, caregiving burden manifests in multiple dimensions, extending beyond psychological distress to disrupt family economic stability, daily functioning, and interpersonal relationships (10). For instance, chronic caregiving responsibilities often lead to economic hardship and a diminished quality of life, creating a vicious cycle that further undermines caregivers’ mental well-being (11). These challenges highlight the complex difficulties faced by caregivers of individuals with mental illness, underscoring the need for greater attention and support (11). Globally, the prevalence of caregiving burden among caregivers of individuals with mental illness is 31.67% (8). In Egypt, the prevalence of caregiving burden among caregivers of individuals with mental illness is 79.19% (12), 55.22% in Nigeria (12), and 41.4% in Jordan (13). Although existing studies indicate cross-national variability in prevalence rates, evidence regarding the prevalence of caregiving burden among family members of individuals with mental disorders in Mainland China remains absent.

Besides, these disparities underscore that the perception and determinants of caregiver burden are not uniform but are profoundly shaped by socio-cultural contexts, including racial, cultural, and systemic factors (14). For example, in European and North American countries, caregiving burden is closely related to the adequacy of the social support system, with notable gender disparities, as female caregivers experience significantly higher rates of depressive symptoms (8, 15, 16). Contrastingly, South African contexts reveal burden primarily arising from mental health literacy deficits and coping skill deficiencies (17). Asian populations demonstrate distinct patterns characterized by pronounced familial obligation and collectivist values influencing burden perception (18).

In China, only one study conducted in Hong Kong identified patient behaviors and mood symptoms as key sources of caregiving burden. (19). However, as Hong Kong is a special administrative region with a distinct sociocultural context shaped by multiple influences, the findings may not fully reflect the circumstances of caregivers in Mainland China. The lack of comprehensive data on burden prevalence and culturally specific analyses of its determinants substantially limits the accurate assessment of caregivers’ unmet needs and the formulation of evidence-based policies in Mainland China. (20, 21). This ongoing research gap perpetuates unmet support needs, undermining both caregiver well-being and the effectiveness of systemic interventions (22).

As the first large-scale survey conducted in Mainland China, this study utilized the Zarit Burden Interview (ZBI) and the Family Burden Scale of Disease (FBSD) to comprehensively assess the prevalence of caregiving burden and identify key related factors among relatives providing care for patients with mental illness. This result helps to improve understanding of the mental health status of patients with mental disorders and provides a basis for further attention to and intervention in the mental health of this population.

## 2 Methods

### 2.1 Study subjects and setting

This cross-sectional study employed a consecutive convenience sampling method to recruit family caregivers of individuals with mental illness from two institutions in Guangzhou, China. To ensure standardized recruitment, caregivers were approached either in the psychiatric inpatient ward of the Affiliated Brain Hospital of Guangzhou Medical University, a tertiary specialized psychiatric hospital, or in the psychiatric outpatient clinics of the Second Affiliated Hospital of Guangzhou Medical University, a tertiary general hospital. Recruitment took place in the inpatient caregivers’ consultation area and the outpatient waiting areas, where eligible caregivers were identified by treating clinicians and invited by trained research staff to participate. Data collection occurred between January and December 2024. A total of 428 caregivers completed the survey. Among them, 28 caregivers were excluded due to incomplete data. Consequently, 400 caregivers met the inclusion criteria and were included in the final analysis. The study protocol received ethical approval from the Institutional Review Boards of the Affiliated Brain Hospital of Guangzhou Medical University (Approval: 2024019). All eligible caregivers provided written informed consent after receiving complete study information. The participants in this study were principal family members providing care for patients with mental illness in Guangzhou, China. Eligibility for inclusion was determined based on predefined selection criteria: (1) aged 18 years or older; (2) having a relative diagnosed with a mental illness by a psychiatrist according to the International Classification of Diseases, Tenth Revision (ICD-10) and currently in an active phase of illness; (3) serving as the primary caregiver, meaning being responsible for daily caregiving duties. Exclusion criteria were as follows: (1) caregivers with severe physical or mental illnesses; (2) caregivers unable to comprehend or complete the required assessment scales. No formal *a priori* sample size calculation was conducted because the study used consecutive sampling in clinical settings. However, with 229 caregivers classified as having burden and six predictors included in the multivariable logistic regression model, the sample size meets the commonly recommended requirement of at least 10 outcome events per predictor variable, indicating adequate statistical power for the planned analyses.

### 2.2 Data assessment

This study employed structured questionnaires to systematically collect demographic and caregiving-related data from all participants. All questionnaires were administered through standardized face-to-face interviews using the validated Chinese versions of the ZBI and FBSD. Interviews were conducted by trained clinicians and required approximately 10–15 minutes to complete. The data included variables such as gender, age, years of education, caregiver-patient relationship, total caregiving duration, number of co-caregivers, primary caregiving reasons, living arrangement, and economic burden pressure from medical expenses. Two clinicians underwent standardized training before data collection. Inter-rater reliability was assessed in a subsample of caregivers using intraclass correlation coefficients (ICC). Agreement was good for both the ZBI (ICC > 0.80) and the FBSD (ICC > 0.80), indicating satisfactory scoring consistency across raters.

Family burden was assessed using the Family Burden Scale of Disease (FBSD). This 24-item scale evaluates six dimensions: (1) Economic burden (6 items); (2) Family activities (5 items); (3) Family entertainment (4 items); (4) Family relationship (5 items); (5) Family member physical health (2 items); (6) Family member mental health (1 item). Higher scores in these six dimensions indicate a greater disease impact on the family. The caregiver burden levels were assessed using the validated Zarit Burden Interview (ZBI) scale, which contains 22 items designed to measure burden intensity, with higher numerical values indicating a more pronounced caregiving burden. Based on established cutoff scores: (1) caregivers with ZBI scores ≥21 were classified as having caregiving burden; (2) caregivers with scores ≤20 were classified as not having caregiving burden. The prevalence of caregiving burden was then calculated based on these classifications (23). Psychometric properties of the FBSD were evaluated in this sample. The total scale demonstrated excellent internal consistency, with a Cronbach’s alpha of 0.93. Alpha coefficients for the six subscales ranged from 0.61 to 0.90, indicating acceptable to excellent internal reliability. All item–total correlations exceeded 0.30, supporting adequate item discrimination.

### 2.3 Data analysis

This study conducted all statistical analyses using IBM SPSS Statistics software, version 27.0.1. The prevalence of caregiving burden was reported as percentages. The prevalence of caregiving burden was reported as percentages. Normality of continuous variables was assessed using the Kolmogorov–Smirnov test. Continuous variables were found not to follow a normal distribution, and therefore non-parametric tests were applied for group comparisons. The descriptive statistics were as follows: (1) categorical variables (gender, caregiver-patient relationship, living arrangement, weekly caregiving hours, co-caregivers, primary caregiving reasons, weekly contact time with patient, economic burden pressure) were presented as frequencies (percentages); (2) continuous variables (age, years of education, total caregiving duration, FBSD scores) were expressed as median (interquartile range, IQR). Comparative analysis involved: (1) chi-square tests for categorical variables; (2) Mann–Whitney U tests for continuous variables, comparing demographic characteristics between the burden group (ZBI score≥21) and the non-burden group (ZBI score ≤ 20). Multivariate analysis included: (1) binary logistic regression to analyze the association between significant variables and caregiving burden, with results expressed as odds ratios (OR) and 95% confidence intervals (CI); (2) receiver operating characteristic (ROC) curve analysis to evaluate predictive factors for caregiving burden, calculating the area under the curve (AUC) with 95% confidence intervals (CI). Statistical thresholds were defined as follows: (1) all tests were two-tailed; (2) predictive relevance required AUC >0.70; (3) statistical significance was set at *p* < 0.05.

## 3 Results

### 3.1 Demographic characteristics

This study included a total of 400 caregivers, and the assessment revealed a caregiving burden prevalence rate of 57.25% in the study population. Compared with the no-burden group, caregivers with burden had a significantly longer total caregiving duration (14(5.0-60.0) vs 30(12.0-150.0) hours, *p* < 0.001), have less co-caregivers (68,8% vs. 57.9%, reporting “No caregivers”, *p* = 0.028), and reported higher levels of economic burden pressure (21.4% vs. 7.6% reporting “hard to bear”, *p* < 0.001). They also scored higher on all six FBSD subscales, reflecting greater impacts on economic burden, family activities, family entertainment, family relationships, and the physical and mental health of family members (all *p* < 0.001). In contrast, age, gender, years of education, caregiver–patient relationship, living arrangement, weekly caregiving hours, weekly contact time, and primary caregiving reasons did not differ significantly between the two groups, indicating that demographic and caregiving characteristics were generally comparable (all *p* > 0.05), as detailed in **Table 1**.

**Table 1:** Comparison of socio-demographics and family burden scale scores between caregivers with caregiving burden and without caregiving burden.

Variables	No burden group (n=171, 42.75%)	Burden group (n=229, 57.25%)	*p*
Gender			
Man, n (%)	62(36.3)	76(33.2)	0.523
Woman, n (%)	109(63.7)	153(66.8)	
Age, median (IQR)	49(44.0-55.0)	48(45.0-53.0)	0.257
Years of education, median (IQR)	12(9.0-15.0)	12(9.0-15.0)	0.988
CPR			
Immediate relatives, n (%)	135(78.9)	194(84.7)	0.135
Other relatives, n (%)	36(21.1)	35(15.3)	
Living arrangement			
LP, n (%)	22(12.9)	32(14.0)	0.489
NLP, n (%)	149(87.1)	197(86.0)	
WCH			
3 days or less, n (%)	36(21.1)	48(21.0)	0.982
3 to 7 days, n (%)	135(78.9)	181(79.0)	
TCD, median (IQR)	14(5.0-60.0)	30(12.0-150.0)	<0.001
Co-caregivers			
Have co-caregivers, n (%)	72(42.1)	72(31.4)	0.028
No co-caregivers, n (%)	99(57.9)	157(68.6)	
PCR			
Voluntary care, n (%)	163(95.3)	215(93.9)	0.758
Forced to take care, n (%)	1(0.6)	1(0.4)	
Substitute family care, n (%)	7(4.1)	13(5.7)	
WCTP			
3 days or less, n (%)	45(26.3)	59(25.8)	0.901
3 to 7 days, n (%)	126(73.7)	170(74.2)	
EBP			
No, n (%)	64(37.4)	28(12.2)	<0.001
Affordable, n (%)	94(55.0)	152(66.4)	
Hard to bear, n (%)	13(7.6)	49(21.4)	
FBSD			
Economic burden, median (IQR)	2(0.0-5.0)	5(3.0-7.5)	<0.001
Family activities, median (IQR)	1(0.0-3.0)	4(2.0-6.0)	<0.001
Family entertainment, median (IQR)	0(0.0-2.0)	3(1.0-4.0)	<0.001
Family relationship, median (IQR)	1(0.0-2.0)	3(2.0-4.5)	<0.001
FMPH, median (IQR)	0(0.0-0.0)	1(0.0-2.0)	<0.001
FMMH, median (IQR)	0(0.0-1.0)	1(0.0-2.0)	<0.001
DFB, median (IQR)	6(2.0-10.0)	17(11.5-23.0)	<0.001

CPR, caregiver-patient relationship; WCH, weekly caregiving hours; TCD, total caregiving duration; PCR, primary caregiving reasons, EBP, economic burden pressure; FMPH, family member physical health; FMMH, family member mental health; DFB, disease family burden; LP, Living with the patient; NLP, Not living with the patient; WCTP, Weekly contact time with patient.

FBSD subscales: Economic burden; Family activities; Family entertainment; Family relationship; FMPH=family member physical health; FMMH=family member mental health.

*p*-values standardized to three decimal places.

### 3.2 Factors associated with caregiving burden

To examine potential determinants influencing caregiving burden levels, our study used a binomial logistic regression approach. The results revealed that increased odds of caregiving burden were associated with the degree of disease impact on family members’ mental health (OR = 2.42, 95% CI: 1.18–4.97), family entertainment (OR = 2.54, 95% CI: 1.24–5.21), family members’ physical health (OR = 2.86, 95% CI: 1.17–7.02), economic burden (OR = 3.32, 95% CI: 1.90–5.78), and family activities (OR = 3.55, 95% CI: 1.61–7.80). In contrast, reduced odds of caregiving burden was associated with having co-caregivers (OR = 0.57, 95% CI: 0.33–0.96), as detailed in **Table 2**.

**Table 2:** Factors associated with caregiving burden.

Variables	OR	95% CI	*p*
Economic burden	3.32	1.90-5.78	<0.001
Family activities	3.55	1.61-7.80	0.002
Family entertainment	2.54	1.24-5.21	0.011
Family member physical health	2.86	1.17-7.02	0.022
Family member mental health	2.42	1.18-4.97	0.016
Co-caregivers	0.57	0.33-0.96	0.036

Variables in the model: (total cargiving duration, economic burden pressure, co-caregivers, economic burden, family activities, family entertainment, family member physical health, family member mental health).

All predictors represent FBSD subscale scores except for “Co-caregivers.” Higher subscale scores indicate greater family burden. For co-caregivers, “Noco-caregivers” serves as the reference category.

Odds ratios (OR) represent the change in the odds of caregiving burden for each 1-point increase in the corresponding subscale score.

### 3.3 Predictive capacity of related factors

To further assess the predictive performance of the identified determinants, our research team conducted an analysis using receiver operating characteristic (ROC) curves. The results indicated that disruptions in family activities (AUC = 0.79), impairments in family entertainment (AUC = 0.79), and economic burden (AUC = 0.76) all demonstrated significant predictive value. Family member physical health (AUC = 0.70) showed acceptable discrimination, whereas family member mental health (AUC = 0.69) approached the acceptable threshold, as detailed in **Table 3**.

**Table 3:** Predictive capacity of associated factors.

Variables	AUC	95%CI
Economic burden	0.76	0.71-0.81
Family activities	0.79	0.74-0.83
Family entertainment	0.79	0.75-0.84
Family member physical health	0.70	0.65-0.75
Family member mental health	0.69	0.64-0.75

## 4 Discussion

This study is the first investigation conducted in Mainland China to assess both the prevalence of caregiving burden among individuals caring for relatives with active-phase mental illness and its distinct associations with caregiver characteristics and family burden. The key findings revealed: (1) the caregiving burden prevalence rate of 57.25%; (2) significant associations between caregiving burden and economic burden, disruption of family activities, impairment of family entertainment, physical health of family members, psychological impact on family members, while the presence of co-caregivers was associated with reduced odds of caregiving burden; (3) the predictive value of disruption of family activities, impairment of family entertainment, and economic burden for the development of caregiving burden.

This study revealed that among family caregivers of individuals with mental illness in China, the prevalence of caregiving burden was 57.25%. This rate is substantially higher than the global average of 31.67% (8) and the European regional rate of 34.3% (24), but relatively lower than the reported rates of 72.0% in India (25) and 87.1% in Iran (26). These regional disparities in the prevalence of caregiving burden can be explained through three key dimensions. Firstly, methodological approaches play a key role. Our study used the Zarit Burden Interview to assess caregiving burden, while studies from different countries utilized various evaluation tools. Previous research has demonstrated that studies using the Zarit Burden Interview tend to report higher burden prevalence compared to those using other assessment instruments (8, 27). Secondly, cultural and perceptual factors significantly influence the assessment of caregiving burden, as there are considerable differences in societal attitudes toward mental illness across regions. Some cultures tend to downplay the severity of mental health issues (28, 29), while China’s Confucian cultural context tends to reinforce stigma, potentially intensifying the caregiving burden (30). Thirdly, disparities in social support systems significantly influence the prevalence of caregiving burden. Walke et al.’s study in India found that 40.9% of caregivers experienced severe burden, primarily due to economic stress, lack of professional support, and social stigma (31). Conversely, the review by Adelman et al. and the study by Gutiérrez-Sánchez et al. demonstrated that robust social support systems can substantially alleviate caregiving burden (32, 33). Wong et al.’s study revealed that family members predominantly care for individuals with schizophrenia. The lack of adequate community mental health services makes it particularly challenging for informal caregivers to manage patients’ behavioral issues and ensure treatment adherence, significantly increasing their psychological burden (34). In summary, the high but inconsistent prevalence of caregiving burden across studies is likely attributable to variations in research methodologies, cultural and regional differences, and the availability of social support systems. These findings underscore the need for interventions that are culturally appropriate and region-specific to effectively address the issue of caregiving burden.

Through further analysis, our study represents the first comprehensive investigation in China to explore the multidimensional associations between caregiving burden and various factors including economic burden (OR = 3.32), disruption of daily family activities (OR = 3.55), impairment of family entertainment (OR = 2.54), physical impacts on family members (OR = 2.86) and psychological impacts on family members (OR = 2.42). Notably, the presence of co-caregivers served as a protective factor, reducing the likelihood of experiencing caregiving burden (OR = 0.57). From the economic burden perspective, long-term treatment of mental illness imposes substantial economic pressure on families (35, 36). Our findings confirm that an increased economic burden significantly intensifies caregiver burden, particularly when treatment costs force families to compromise on essential household needs. For instance, families may reduce spending on necessities to afford expensive treatments, further compromising daily living standards and exacerbating caregivers’ psychological stress and burden (37). Additionally, research has shown that the economic burden on families caring for individuals with mental illnesses extends beyond direct medical costs to include reduced income and increased living expenses (38). For instance, caregivers may need to reduce work hours or even quit their jobs to provide care, resulting in a significant loss of income (39). Finally, the costs associated with caregiving-such as transportation, medication, and consultations-further strain the family’s economic resources (40). Regarding the disruption of family activities, the presence of mentally ill family members often interrupts household routines (41, 42). Moreover, caregivers must dedicate considerable time and energy to assisting patients with daily living activities, such as personal care, meals, housework, shopping, and transportation (43). Our results align with this observation, showing a strong positive correlation between the extent of family activity disruption and caregiving burden. Regarding family entertainment, previous study found that reduced recreational activities are closely associated with caregiving burden in families managing chronic illness (44). Our findings confirm this relationship, showing significant positive correlations between the impairment of family entertainment and caregiving burden. Regarding physical health consequences, a meta-analysis by Pinquart and Sörensen (2007) revealed that long-term caregiving stress negatively impacts caregivers’ physical health, including weakened immune function and increased cardiovascular risks (45). Our results similarly indicate that the deteriorating physical health of family members exacerbates caregiver stress and burden. At the psychological level, Jeyagurunathan et al. found that family members of individuals with mental illness often experience anxiety and depression due to prolonged exposure to patient symptoms and concerns about prognosis (46). Our study confirms that caregivers must manage both the patient’s condition and the psychological states of other family members, significantly increasing their burden. Our findings also underscore the protective role of co-caregivers. Caregivers who received assistance from co-caregivers were less likely to experience high caregiving burden, which is consistent with previous research. Liu et al. and Applebaum and Sannes reported that shared caregiving reduces strain and fatigue by distributing responsibilities across multiple individuals (47, 48). Peng et al. similarly demonstrated that the availability of co-caregivers, providing both instrumental and emotional support, significantly decreases the likelihood of developing a heavy caregiving burden (20). These findings collectively emphasize the critical need for shared caregiving support systems. Our results not only highlight the profound impact of mental illness on families but also offer new perspectives on understanding the complexity of caregiving burden within the Chinese context.

Furthermore, this study is the first to demonstrate that the degree of disruption in family activities (AUC = 0.79), impairment of family entertainment (AUC = 0.79), and economic burden have (AUC = 0.76) predictive value for caregiving burden—findings that have been substantiated by multiple studies. Tomoko Omiya et al. and Mie Sedoc Jørgensen et al. reported that the extent of restrictions in both family activities and family entertainment was positively correlated with caregiving burden (49, 50). These studies collectively indicate that significant disruptions to family and recreational activities substantially increase caregiving burden. Rao et al. and Philip S. Wang et al. further emphasized that severe economic strain not only undermines household financial stability but also induces caregiving burden and feelings of helplessness in caregivers, thereby exacerbating their overall burden (51, 52). These findings underscore the critical importance of providing caregivers with adequate support and resources to alleviate economic pressures and sustain balanced family and recreational activities-key measures for effectively reducing caregiving burden. The evidence highlights the need for comprehensive interventions that address both the socioeconomic and psychosocial dimensions of caregiving challenges. From a psychological perspective, these outcomes may be linked to caregivers’ psychological resilience. Caregivers with lower resilience may find it more difficult to cope with restrictions in daily and leisure activities, thereby experiencing a greater caregiving burden (53). Additionally, economic burden can contribute to anxiety and depression in caregivers, and these negative emotions may further weaken their psychological resilience, thereby increasing the caregiving burden (54). Therefore, providing caregivers with sufficient support and resources, alleviating economic pressures, and helping them maintain a balance between daily family responsibilities and leisure activities are crucial for reducing caregiving burden.

The findings of this study should be interpreted in the context of its limitations. First, the sample included 400 caregivers of mental illness patients in Guangzhou, limiting the findings’ generalizability to other regions or types. Second, the study did not collect data on caregivers’ psychological resilience and coping strategies, which could significantly influence caregiving burden. Lastly, the study lacked information on the specific diagnoses and treatment plans of the patients, which might affect the comprehensive understanding of the factors influencing caregiving burden.

In summary, this study is the first to systematically explore the prevalence of caregiving burden and its association with personal and family circumstances among relatives providing care for patients with mental illness in Mainland China. The study results showed that the prevalence of caregiving burden was 57.25%. Caregivers who received support from co-caregivers were less likely to experience caregiving burden, whereas higher economic burden was associated with an increased likelihood of burden. Additionally, the impact of the patient’s illness on family activities, family entertainment, and the physical and mental health of family members was more pronounced in caregivers with burden than in those without burden. These findings establishe restoring family function as a core target, offering a practical route to low-cost interventions in collectivist societies.Future research should adopt longitudinal study designs and include more individual-difference variables and patient-related information to further validate the conclusions of this study and explore the mechanisms influencing caregiving burden.

## Data Availability

The raw data supporting the conclusions of this article will be made available by the authors, without undue reservation.
